# A national description of emergency medical services patient utilization patterns

**DOI:** 10.1093/haschl/qxag144

**Published:** 2026-06-08

**Authors:** Antonio R Fernandez, Jonathan Brent Myers, Scott S Bourn, Remle P Crowe, Edward C Preusser, James Muisyo, Alison Treichel, Alyssa M Green, Kehinde Onaeko, Eric H Beck

**Affiliations:** ESO, Inc., Austin, TX78722, United States; Department of Emergency Medicine, School of Medicine, University of North Carolina at Chapel Hill, Chapel Hill, NC27599, United States; ESO, Inc., Austin, TX78722, United States; ESO, Inc., Austin, TX78722, United States; ESO, Inc., Austin, TX78722, United States; Department of Emergency Health Sciences, University of Texas Health San Antonio, San Antonio, TX 78201, United States; ESO, Inc., Austin, TX78722, United States; ESO, Inc., Austin, TX78722, United States; ESO, Inc., Austin, TX78722, United States; ESO, Inc., Austin, TX78722, United States; ESO, Inc., Austin, TX78722, United States; ESO, Inc., Austin, TX78722, United States; Department of Emergency Medicine, University Hospitals Cleveland Medical Center, Case Western Reserve University School of Medicine, Cleveland, OH44106, United States

**Keywords:** emergency medical services, high utilization, frequent 911 use, health care utilization, Medicaid, social vulnerability, community paramedicine, prehospital care, health equity, care coordination, alternative payment models, population health, social determinants of health, EMS data, mobile integrated health

## Abstract

**Introduction:**

Repeat use of 9-1-1 emergency medical services (EMS) reflects complex intersections of medical need, social vulnerability, and health system failures. While high utilization of emergency departments has been extensively studied, national characterizations of EMS utilization patterns remain limited.

**Methods:**

We conducted a retrospective analysis of 9 508 809 EMS encounters involving 6 604 938 unique patients using the 2024 ESO Data Collaborative. A novel patient-level linkage approach using the Levenshtein distance method identified repeat encounters across time. EMS utilization was categorized as low (1), moderate (2-4), high (5-11), and very-high (12+ encounters).

**Results:**

Three percent of patients accounted for 16% of all encounters. Patients with very-high utilization were disproportionately covered by Medicaid, resided in socioeconomically vulnerable communities, and more commonly presented with chronic conditions. Despite higher transport rates, they were 40% less likely to be admitted than single-encounter patients. The median time between encounters was 10 days for very-high utilizers.

**Conclusion:**

These patterns are consistent with broad health and healthcare access challenges rather than individual patient behavior. EMS utilization data may offer a means to identify high-need, low-access populations and develop strategies that improve care coordination and reduce emergency system strain.

**Key points**

- A small subset of patients—approximately 3%—accounted for 16% of all 911 EMS encounters in 2024, with very-high utilizers returning to EMS care within a median of 10 days.
- Patients with very-high utilization were disproportionately covered by Medicaid and resided in socioeconomically vulnerable communities, suggesting that frequent EMS use reflects structural barriers to care rather than individual behavior.
- EMS utilization data offer an underused early signal for identifying high-need populations, and policy actions such as expanded community paramedicine reimbursement and alternative payment models warrant serious consideration.

## Introduction

Patients who repeatedly access emergency care represent one of the most complex, clinically vulnerable, and policy-relevant populations in the US healthcare system. Commonly defined as patients with 4 or more emergency department (ED) visits within a 12-month period, patients with high utilization frequently present with overlapping medical, behavioral, and social needs.^1-4^ Mental health conditions and substance use disorders are prevalent, often alongside chronic illnesses.^1,5^ Many face substantial barriers to accessing care and experience socioeconomic vulnerability,^4^ disabilities, or housing insecurity. Despite representing 4%-16% of ED patients,^1,6^ patients with high utilization account for 14%-47% of ED visits, contributing to system strain, costs, crowding, and reduced availability of resources for time-sensitive care.^4,7,8^

Emergency medical services (EMS) occupy a pivotal but underexamined role in care experiences for patients with high utilization. EMS represents the first, and sometimes only, healthcare contact for many with limited access to primary, specialty, behavioral health, and other essential services. Critically, neither EMS nor the ED was designed to provide the longitudinal, integrated care these patients need. Yet for many, it remains the most consistently accessible point of entry. Between 46% and 84% of frequent ED patients arrive via ambulance,^9-12^ compared with 20% of all ED patients.^13^

Although the US EMS system was designed to deliver rapid response for acute emergencies,^13,14^ a large proportion of 9-1-1 calls involve chronic or lower-acuity presentations, reflecting a broader pattern in which EMS and the ED serve as de facto entry points for patients with limited access to the health system.^7^ EMS has its own frequent users, often defined as patients with 5 or more encounters per year.^1,9,15,16^ These patients account for a disproportionate share of EMS responses and contribute substantially to ED utilization and healthcare spending.^9^

Despite EMS's ability to identify patients with unmet needs early and connect them to care pathways, EMS data have been underrepresented in health services research and largely absent from national policy discussions. Existing studies of frequent EMS users have been limited in scale, geography, or scope, relying on small samples or narrow populations.^12^ A 2014 systematic review found that frequent EMS users accounted for a disproportionate share of transports despite representing a small minority of patients; however, most studies were inpatient-focused with limited EMS data.^12^ The Inspector General of the US Department of Health and Human Services reported that Medicare spending on ambulance transport increased 130% between 2002 and 2011 despite only 7% increase in Medicare recipients; EMS frequent users could have contributed to that increase.^17^ Yet these studies provide limited insight into patient characteristics, patterns, and intervention opportunities nationally.

Understanding frequent EMS utilization is increasingly important as policymakers pursue strategies to improve care for patients with unmet needs, reduce avoidable emergency care, expand alternative response models, and control costs. EMS data offer a uniquely early, community-based view of patient needs and represent a critical but underused resource for population health management. This study characterized repeat EMS encounters across a large national dataset and identified patient and encounter-level factors related to high utilization.

## Study data and methods

### Study design and data source

This was a retrospective analysis of 9-1-1 EMS encounters using the 2024 ESO Data Collaborative, a multijurisdictional dataset from a national prehospital electronic health record system (ESO, Inc.; www.eso.com/data-and-research). ESO software supports the standardized collection of comprehensive EMS information, including dispatch characteristics, patient demographics, clinical assessments, treatments, and dispositions across more than 3000 participating agencies in the United States.

Historically, EMS data have been structured as discrete encounter-level records, limiting examination of patient-level utilization patterns. This constraint has posed a significant barrier to understanding repeat EMS use, longitudinal care trajectories, and opportunities for early intervention. To address this limitation, we implemented a novel patient-level linkage approach enabling the identification of repeat encounters attributable to the same individual across time while preserving privacy.

All 9-1-1 responses with patient contact between January 1 and December 31, 2024, were eligible for inclusion. To link encounters belonging to the same individual, a masked patient ID was generated by comparing primary and secondary demographic fields (including name and date of birth) using the Levenshtein distance method. This approach calculates the minimum number of single-character edits required to transform one string into another, enabling probabilistic matching of records that may contain minor typographical variations or data entry inconsistencies across encounters. Match thresholds were established to balance sensitivity and specificity. Records with EMS dispositions documented as assist, standby, or canceled were excluded, as these do not represent patient care interactions. Records lacking sufficient demographic information to support reliable linkage were also excluded. Finally, duplicate records for multiunit response, identified by repeated incident numbers, were removed prior to analysis.

St. David's HealthCare IRB determined this study exempt.

### Measures

Our primary variable of interest, EMS utilization, was categorized based on the number of encounters within the 12-month study period: low (1), moderate (2-4), high (5-11), and very-high (12+ encounters). Patient demographics included age (adult [18-64], older adults [≥65], adolescent [13-17], school age [5-12], toddler/preschool [1-4], baby [<1]), sex (male/female), and race/ethnicity (American Indian/Alaskan Native, Asian, Black/African American, Native Hawaiian/Pacific Islander, Hispanic/Latino, Multiracial, White; all except Hispanic/Latino are not Hispanic/Latino).

Clinical impressions were collapsed into 11 clinically meaningful categories (Appendix Table A1). Clinical severity was assessed using the Rapid Emergency Medicine Score (REMS), a validated prehospital score.^18^ REMS was calculated using the initial vital signs from the index encounter. Socioeconomic status of the incident location was evaluated using the CDC Social Vulnerability Index (SVI).^19^ Community size was characterized using the Geographic Area Definitions described in the CMS Ambulance Fee Schedule.^20^ EMS transport status was derived from the prehospital disposition field as transported by EMS or not. For patients with repeat utilization, the number of days between the index EMS encounter and the second EMS encounter was calculated. For encounters that resulted in EMS transport with linked ED data, we described payer categories, ED disposition, and for patients who were admitted, hospital disposition. The payer category for the EMS event was classified as commercial insurance, Medicaid, Medicare, Veterans Affairs, workers’ compensation, self-pay, multiple payers, or other. Patients with more than one EMS encounter during the study period, and more than one documented payer type had a payer category defined as multiple payers.

### Data analysis

Patient demographics and clinical characteristics were summarized at patient-level; incident-level variables at encounter level. Univariable odds ratios (OR) and 95% confidence intervals (CI) were calculated at the patient-level for evaluating the relationship between demographic, clinical presentation and the odds of very-high EMS utilization compared to one EMS encounter in the calendar year. Similarly, the odds of very-high EMS utilization were calculated at the encounter level for incident location and community size. Because accurate assessment of payer status required linkage to hospital records, analyses involving payer status were restricted to encounters with available hospital outcomes. In addition, the odds of hospital admission (discharged vs admitted) were calculated across all 4 EMS utilization categories and all 8 payer categories. All analyses were performed using Stata v18 (College Station, TX).

## Results

In 2024, 12 568 565 9-1-1 responses were documented in the ESO Data Collaborative. After excluding canceled calls (*n* = 1 199 569), records missing linkage demographics (*n* = 1 256 215), non-patient-care dispositions (eg, assist, standby; *n* = 142 489), and multiunit responses (*n* = 461 483), the analytic sample included 9 508 809 EMS encounters involving 6 604 938 unique patients (Table 1).

**Table 1.** EMS utilization patterns by patient and encounter.

Number of 9-1-1 encounters in 2024	Unique patients in category (col %)	9-1-1 encounters (col %)
1 encounter	81.21% (5 363 570)	56.41% (5 363 570)
2-4 encounters	15.95% (1 053 765)	27.21% (2 587 480)
5-11 encounters	2.46% (162 734)	11.14% (1 059 250)
12+ encounters	0.38% (24 869)	5.24% (498 509)
Total	100.00% (6 604 938)	100.00% (9 508 809)

Most patients (81%, *n* = 5 363 570) had one encounter, accounting for 56% of responses. A total of 16% (*n* = 1 053 765) of patients had moderate utilization (2-4 encounters), representing 27% of encounters (*n* = 2 587 480). Patients with high utilization (5-11 encounters) represented 2% of patients (*n* = 162 734) and 11% of encounters (*n* = 1 059 250). Patients with very-high utilization (≥12 encounters) comprised 0.4% of patients (*n* = 24 869) yet accounted for 5% of encounters (Table 1).

### Demographic and clinical characteristics

Demographic and clinical characteristics varied meaningfully across utilization groups (Table 2). Most patients with very-high utilization were adults aged 18-64 years (63%), and over one-third were ≥65 years old (36%). Compared to the overall cohort, a higher proportion of those with very-high utilization were characterized as Black or African American (26%) or Multiracial (12%).

**Table 2.** Patient and clinical characteristics by EMS utilization category.

Variable % (*n*)	All patients	Patients with 1 encounter	Patients with 2-4 encounters	Patients with 5-11 encounters	Patients with 12+ encounters
**Total population**	100.00% (6 604 938)	81.21% (5 363 570)	15.95% (1 053 765)	2.46% (162 734)	0.38% (24 869)
**Sex**					
Female	51.94% (3 412 170)	51.45% (2 749 455)	54.31% (566 156)	53.25% (84 836)	49.23% (11 723)
Male	48.06% (3 156 977)	48.55% (2 594 104)	45.69% (476 305)	46.75% (74 479)	50.77% (12 089)
Race/ethnicity*					
American Indian/Alaskan Native	0.45% (24 334)	0.48% (20 680)	0.32% (3103)	0.31% (476)	0.32% (75)
Asian	1.76% (96 027)	2.01% (86 960)	0.88% (8426)	0.40% (609)	0.14% (32)
Black or African American	20.10% (1 096 704)	20.20% (872 591)	19.27% (185 145)	21.41% (32 930)	26.14% (6038)
Native Hawaiian or other Pacific Islander	0.23% (12 644)	0.27% (11 641)	0.10% (929)	0.04% (67)	0.03% (7)
Hispanic/Latino	10.14% (553 317)	11.39% (491 925)	5.60% (53 777)	4.35% (6693)	3.99% (922)
Multiracial	2.90% (158 467)	2.31% (99 812)	4.62% (44 406)	7.52% (11 562)	11.63% (2687)
White	64.42% (3 515 567)	63.34% (2 735 831)	69.21% (664 957)	65.97% (101 440)	57.74% (13 339)
**Age categories**					
Baby (<1 year)	1.42% (93 481)	1.63% (87 374)	0.56% (5895)	0.13% (205)	0.03% (7)
Toddler/preschool (1-4)	1.09% (72 037)	1.28% (68 392)	0.33% (3488)	0.09% (149)	0.03% (8)
School age (5-12)	2.18% (143 493)	2.53% (135 709)	0.69% (7236)	0.32% (514)	0.14% (34)
Adolescent (13-17)	3.19% (210 190)	3.63% (194 590)	1.38% (14 471)	0.66% (1052)	0.32% (77)
Adult (18-64)	52.47% (3 459 888)	54.99% (2 949 382)	40.59% (424 734)	43.91% (70 355)	63.39% (15 417)
Older Adults (≥65)	39.66% (2 615 339)	35.95% (1 928 123)	56.43% (590 473)	54.90% (87 967)	36.09% (8776)
**Index visit clinical impression**					
Cardio/pulmonary	15.50% (1 023 710)	14.69% (787 949)	18.86% (198 688)	19.85% (32 310)	19.15% (4763)
AMS/behavioral/psych	13.54% (894 355)	13.65% (732 090)	13.07% (137 715)	12.75% (20 745)	3.16% (786)
CNS/Neuro	5.36% (353 897)	4.80% (257 425)	7.81% (82 321)	7.80% (12 699)	5.48% (1364)
Seizure	2.90% (191 552)	2.84% (152 207)	3.17% (33 351)	3.15% (5127)	3.49% (867)
Diabetic/endocrine	1.41% (93 036)	1.25% (67 212)	1.97% (20 786)	2.61% (4252)	3.16% (786)
Injury/pain	34.48% (2 277 359)	35.53% (1 905 583)	30.21% (318 373)	28.39% (46 207)	28.94% (7196)
Infection/immune	1.88% (124 220)	1.79% (96 154)	2.26% (23 779)	2.30% (3749)	2.16% (538)
GI/GU	4.05% (267 462)	3.76% (201 697)	5.20% (54 825)	5.88% (9576)	5.48% (1364)
OB/GYN	0.60% (39 431)	0.64% (34 208)	0.45% (4765)	0.25% (413)	0.18% (45)
Cardiac arrest	1.05% (69 347)	1.28% (68 620)	0.06% (680)	0.03% (47)	0.00% (0)
Other	19.24% (1 270 476)	19.77% (1 060 355)	16.94% (178 460)	16.97% (27 608)	16.30% (4053)
**Index visit median REMS (IQR)**	5 (2-7)	5 (2-7)	6 (4-8)	6 (4-8)	5 (2-7)
**Days between index and second encounter, median (IQR)**	40.0 (10.4-103.1)	N/A	44.1 (11.6-111.4)	27.9 (8.1-70.5)	10.0 (3.0-26.9)

All race/ethnicity categories except Hispanic/Latino are not Hispanic/Latino.

Clinical impression data based on provider's primary impression at index EMS encounter.

Clinical severity, as measured by the median REMS on the index EMS encounter, was similar among patients with one encounter and those with ≥12 encounters, while modestly higher REMS values were observed for patients with 2-11 encounters. Patterns of repeat utilization differed substantially by utilization category: the median time between the index EMS encounter and the subsequent encounter was 44 days among patients with moderate utilization (2-4 encounters) compared with 10 days among those with very-high utilization (≥12 encounters) (Table 2).

Patients with EMS presentations related to diabetes had nearly twice the odds of very-high utilization (OR: 1.93, 95% CI: 1.79-2.09) vs cardiopulmonary, while GI/GU showed elevated odds (OR: 1.12, 95% CI: 1.05-1.19) (Appendix Table A2).

### Social context and transport

Social vulnerability differed markedly by utilization category. Patients with 5+ encounters accounted for over one-third of responses in the most vulnerable locations (Appendix Table A3). Most vulnerable regions (SVI Quartile 4) had threefold higher odds of very-high utilization vs least vulnerable (Q1) (OR: 2.96, 95% CI: 2.93-2.99) (Table 3). Quartiles 2 and 3 showed progressive increases (OR: 1.55, 2.01), demonstrating a clear vulnerability gradient.

**Table 3.** Encounter-level odds ratios for very-high EMS utilization.

Variable	Univariable odds ratio	95% confidence intervals
**CDC Social Vulnerability Index**		
Quartile 1 (least vulnerable)	Referent	—
Quartile 2	1.55	1.53-1.56
Quartile 3	2.01	1.99-2.03
Quartile 4 (most vulnerable)	2.96	2.93-2.99
**Community size**		
Urban	Referent	—
Rural	1.02	1.01-1.03
Super rural	0.58	0.57-0.60
**EMS transport disposition**		
Transported by EMS	Referent	—
Non-transport	1.57	1.56-1.58
Dead on scene	0.06	0.05-0.07

Notes: Odds ratios compare patients with very-high utilization (12+ encounters) to patients with a single encounter. Analysis conducted at the encounter-level (*n* = 9 508 809).

Patients with very-high utilization were transported more often than single-encounter patients (83% vs 75%) (Appendix Table A3). When compared to urban areas, rural and super-rural areas showed decreased odds of very-high utilization (OR: 1.02 rural, 0.58 super-rural) (Table 3).

### Emergency department and hospital outcomes

ED disposition data were available for 31% of transport encounters (2 345 496/7 521 032). Among patients with very-high utilization, 75% of ED visits resulted in discharge vs 64% for patients with a single encounter (Appendix Table A4). Very-high utilization was associated with 40% reduced hospital admission odds (OR: 0.58, 95% CI: 0.57-0.59). Moderate utilization was associated with elevated admission odds (OR: 1.57, 95% CI: 1.56-1.58) (Table 4).

**Table 4.** Odds ratios for hospital admission by EMS utilization category.

EMS utilization category	Univariable odds ratio	95% confidence intervals
1 encounter	Referent	—
2-4 encounters	1.57	1.56-1.58
5-11 encounters	1.37	1.36-1.38
12+ encounters	0.58	0.57-0.59

Notes: Odds ratios compare odds of hospital admission (vs discharge) across utilization categories. Analysis restricted to encounters with linked ED disposition data (*n* = 2 345 496).

Payer mix differed substantially. Medicaid proportion increased with utilization category: 13% of patients with a single-encounter vs 24% among patients with very-high utilization (Appendix Table A4). Encounters with Medicaid coverage had threefold higher odds of belonging to the group with very-high utilization (OR: 3.11, 95% CI: 3.07-3.13) vs commercial insurance. Encounters with Medicare coverage showed moderate elevation in odds of very-high utilization (OR: 1.62, 95% CI: 1.60-1.64). Encounters with multiple payer types listed also showed elevated odds of very-high utilization (OR: 1.74, 95% CI: 1.72-1.76) (Appendix Table A5). Hospital dispositions mirrored ED patterns, with higher discharge rates among patients with very-high utilization (Appendix Table A4).

## Discussion

Using a large national dataset of 9.5 million EMS encounters involving 6.6 million patients, this study provides one of the most comprehensive characterizations of repeat EMS utilization patterns in the United States. Most patients accessed 9-1-1 once yearly; however, approximately 3% of patients accounted for 16% of encounters, reflecting a striking concentration of EMS utilization among a small subset of the population. Patients with high and very-high utilization exhibited distinct demographic, clinical, and socioeconomic profiles, reinforcing findings from ED-based research while extending them to prehospital settings. Our results underscore EMS as an early, underutilized data source for identifying patients with unmet needs and potentially low access to care.

The concentration parallels findings from ED research, where small groups account for disproportionate visit volumes.^1,6^ Patients with very-high utilization comprised 0.4% of patients yet generated 5% of encounters, and they returned to EMS care rapidly, with a median of only 10 days between encounters. This compressed cycle suggests persistent unmet needs. While prior studies documented high costs associated with frequent utilization,^1^ our findings indicate entrenched patterns of care-seeking among patients who repeatedly turn to EMS, reflecting systemic gaps rather than individual choice.

Contrary to assumptions that patients who frequently use EMS primarily present with low-acuity conditions,^21^ clinical severity, as measured by REMS, was similar between patients with single-encounter and those with very-high utilization.^18^ Yet patients with very-high utilization were significantly less likely to be admitted, with 75% of ED visits resulting in discharge compared to 64% among patients with single encounters. This combination warrants careful interpretation. REMS reflects physiologic acuity at a single point in time and does not capture the complexity of chronic disease burden, social circumstances, or cumulative care needs that may drive repeat utilization. The low admission rate among patients with very-high utilization may reflect ED clinical judgment that acute hospitalization is not indicated, even when underlying needs are substantial and unmet. Together, these patterns suggest that frequent EMS activation is less a marker of episodic acute illness and more a signal of persistent unmet needs within a system not structured to address them longitudinally.

Socioeconomic status emerged as one of the strongest relationships identified for very-high utilization. Patients encountered in the most socioeconomically vulnerable communities showed threefold higher odds of very-high utilization, and those covered by Medicaid demonstrated similarly elevated odds compared to patients with commercial insurance. While our observational design cannot establish causality, these gradients are consistent with structural barriers—limited primary care access, behavioral health services, and care coordination—identified as contributors in prior research.^1,14,16^ Consistent with work emphasizing that frequent emergency use is related to broader social and system failures rather than individual behavior,^4^ our findings are consistent with the view that high emergency utilization reflects broad health and healthcare access challenges, and that meaningful reduction may depend on responses at the system level.

### Policy implications

These results highlight EMS as a critical but underutilized population health asset. EMS encounters often represent a recurring and community-based touchpoint within fragmented care systems, offering opportunities to identify patients with unmet needs and connect them to coordinated care before hospital-based utilization escalates. Frequent transport with low admission rates may indicate an opportunity for alternative response models, including mobile integrated health and community paramedicine, which have shown promise in reducing calls, ED visits, admissions, and costs in prior studies.^8,22-26^ Only 6%-12% of 9-1-1 calls require time-sensitive interventions in the EMS and ED settings,^27^ reinforcing alternative pathway potential.

Specific policy actions include: First, state Medicaid programs should consider expanding community paramedicine reimbursement, following state models covering non-transport encounters for care coordination, medication reconciliation, and primary care referrals. The threefold higher utilization odds among Medicaid beneficiaries suggest that coverage expansions may offer meaningful returns, though formal cost-effectiveness analyses will be needed to quantify the potential impact. Second, accelerating alternative payment models that reward reduced transports rather than volume warrants consideration, as current fee-for-service structures create disincentives for alternative programs. Third, investment in data-sharing infrastructure to enable EMS real-time access to recent utilization, care plans, and community resources warrants consideration. Fourth, policymakers should create incentives for EMS integration with federally qualified health centers and Medicaid managed care organizations, enabling warm handoffs, shared care plans, and follow-up for patients with unmet needs identified through EMS contact. Fifth, explicitly including EMS coordination roles in community health worker funding programs could help bridge prehospital and community-based care for patients with frequent utilization.

The economic implications merit further investigation. Between 2002 and 2011, frequent users were associated with 130% Medicare spending increases despite only 7% beneficiary growth.^17^ While precise national cost estimation is beyond the scope of this study, per-patient costs of $25 000 annually among frequent emergency users.^3^ Prior research has also documented potential savings associated with EMS-based programs for those with frequent utilization patterns, though optimal strategies may vary by community and infrastructure. Dedicated cost-effectiveness analyses would help quantify the potential impact of targeted interventions.

Beyond costs, system capacity implications for both EMS and hospitals are substantial. High utilization contributes to crowding, wait times, reduced time-sensitive resource availability, and decreased workforce satisfaction.^28,29^ It limits surge capacity, reducing resilience during emergencies, disasters, and pandemics.^7^ Managing frequent utilization is thus about efficiency, preparedness, and quality.

### Limitations

Our analysis was restricted to one calendar year, limiting longer-term trajectory assessment and whether patients identified with utilization categories of high or very-high maintain those patterns over time. Single-year analyses may also underestimate true utilization for patients whose high-use periods span calendar year boundaries. Longitudinal studies spanning multiple years would strengthen understanding of utilization stability, escalation, and de-escalation patterns.

The ESO Data Collaborative includes more than 3000 agencies but is not population-representative of all US EMS systems. Participating agencies may systematically differ from non-participants in organizational size, technology adoption, urban–rural distribution, and patient populations served. Findings should therefore be interpreted as characterizing patterns within this large but self-selected sample rather than as nationally representative estimates. That said, ESO represents one of the largest linked EMS-hospital datasets available, and observed patterns are consistent with smaller regional studies, supporting the generalizability of the broad directional findings.^30^

Outcome information was available for 31% of transports, potentially limiting generalizability of ED disposition and payer findings (Appendix Table A5). Linked data were more commonly available for encounters in urban (91.6% vs 77.1%) and those areas of high socioeconomic vulnerability (31.6% vs 28.3%). However, it should be noted that the core findings of this study used the full data regardless of linkage status.

Observational design precludes causal inference. Strong associations between Medicaid, social vulnerability, and frequent use cannot determine direct causation vs markers for unmeasured characteristics. Cross-sectional data prevent temporal relationship assessment.

Levenshtein linkage enabled patient-level analysis but permits false-positive and false-negative linkages. Though some degree of false-positive and false-negative linkage is inevitable with any probabilistic approach, misclassification is expected to bias utilization estimates toward the null, as unlinked encounters would tend to undercount repeat utilization rather than artificially inflate it. Data exclude out-of-jurisdiction encounters and lack information on primary care distance, transportation, and social support.

Despite limitations, findings support the value of patient-level linkage and position EMS data as critical early signals for patient populations with unmet needs. Integrating EMS data into health system planning and payment models represents a promising strategy for reducing avoidable care and improving equity.

## Conclusion

Repeat EMS utilization represents an underused window into populations whose needs remain unmet by the systems intended to serve them. This study's findings indicate that frequent EMS use is concentrated among patients facing chronic diseases and profound socioeconomic challenges, and structural barriers to primary, behavioral, and specialty care. Rapid return intervals, high transport rates, and low admission rates suggest misalignment between patient needs and available pathways. While observational design limits causal inference, strong associations between community socioeconomic vulnerability, Medicaid coverage, and utilization are consistent with structural barriers documented in prior research. Shifting EMS data from encounter-based to patient-centered highlights EMS as an underutilized early signal for identifying populations with unmet needs. As systems and policymakers seek to control costs, address inequities, and preserve capacity for time-sensitive care and emergencies, specific actions such as expanding Medicaid reimbursement for community paramedicine, creating alternative payment models, and investing in data-sharing infrastructure—warrant serious consideration as strategies for improving efficiency, quality, and resilience. The demographic and clinical profiles identified in this study can guide more precise intervention targeting, though rigorous evaluation of effectiveness and cost-effectiveness would be needed to optimize resource allocation and support sustainable improvements.

## Supplementary Material

qxag144_Supplementary_Data

## Data Availability

The ESO Data Collaborative research dataset is publicly available at no cost following submission of a research proposal, completion of a data use agreement, and IRB determination. Research proposals are reviewed by the ESO Research Leadership Group, an external volunteer committee of industry leaders charged with evaluating requests to ensure the data are sufficient to answer the intended question and that appropriate constraints regarding commercial bias and conflicts of interest are maintained. Requests may be submitted at www.eso.com/data-and-research.
