# Supplementary material for: A national description of emergency medical services patient utilization patterns
Source: Health Aff Sch. 2026 Jun 8;4(6):qxag144. doi: 10.1093/haschl/qxag144 (PMC13282441; doi:10.1093/haschl/qxag144)
Supplement: qxag144_Supplementary_Data [file qxag144_supplementary_data.zip › Appendix Materials.docx]

**Online Appendix**

**A National Description of EMS Patient Utilization Patterns**

**Appendix Table A1. Clinical Impression Categories**

**AMS/Behavioral/Psych**

alcohol dependence with withdrawal

alcohol intoxication

alcohol use

altered mental status

altered mental status, transient

anxiety reaction/emotional upset

behavioral/psychiatric episode

cannabis related disorder

cocaine related disorders

coma

concussion with loss of consciousness

confusion/delirium

deliberate self harm

dementia

depression

general psychiatric exam, requested by authority

hallucinogen related disorders

homicidal ideation

mental disorder

opioid related disorders

other personality disorder

other stimulant related disorders

overdose - acetaminophen

overdose - alcohol

overdose - amphetamine

overdose - benzodiazepine

overdose - cannabis

overdose - cocaine

overdose - fentanyl

overdose - hallucinogens

overdose - heroin

overdose - methadone

overdose - opium

overdose - other opioids

overdose - polysubstance

overdose - synthetic marijuana

overdose - synthetic narcotics

overdose - unspecified

overdose - xylazine

poisoning / drug ingestion

sedative, hypnotic, or anxiolytic related disorders

substance abuse

suicidal ideation

suicide attempt

unconscious

**Injury or Pain**

abdominal pain

acute appendicitis

acute pain, not elsewhere classified

amputation of limb

amputation of other parts of head (face, ears,etc.)

back pain

brain injury

burn

chemical burn

chest pain on breathing

chest pain, other (non-cardiac)

concussion

concussion without loss of consciousness

drowning

early complication of trauma

electrocution

epidural hemorrhage

extremity pain

eye injury

eye pain

frostbite, superficial

frostbite, with tissue necrosis

headache

hemorrhage

hemothorax (traumatic)

illness, unspecified

injury

injury of abdomen

injury of ankle

injury of ear

injury of elbow

injury of external genitals

injury of face

injury of foot

injury of forearm

injury of head

injury of hip

injury of lower back

injury of lower leg

injury of neck

injury of nose

injury of pelvis

injury of shoulder or upper arm

injury of thigh (upper leg)

injury of thorax (upper chest)

injury of wrist, hand, or fingers

kidney stones

knee injury

knee pain

laceration (major)

laceration/abrasion/hematoma (minor surface trauma)

limb/hand/foot/fingers/toes pain

lower back pain

migraine

multiple injuries

obstetric trauma

pain (non-traumatic)

pain, acute due to trauma

pain, chronic

pelvic and perineal pain

pneumothorax (traumatic)

rhabdomyolysis

sexual abuse

sickle cell crisis

soft tissue wound

subarachnoid hemorrhage (traumatic)

subdural hemorrhage (traumatic)

toothache

traumatic circulatory arrest

wound care

**Cardio/Pulmonary**

acute bronchospasm

acute coronary syndrome

acute respiratory distress (dyspnea)

anaphylactic shock

anaphylaxis

anemia

angina pectoris

angina, unstable

asthma

atelectasis

carbon monoxide poisoning

cardiac arrhythmia/dysrhythmia

cardiac tamponade

cardiogenic shock

chest pain / discomfort

chronic obstructive pulmonary disease (copd)

congestive heart failure (chf)

deep vein thrombosis - dvt - (blood clot)

foreign body in nostril

foreign body in pharynx

foreign body in respiratory tract

foreign body in trachea

heatstroke and sunstroke

hemorrhagic shock

hyperkalemia

hypertensive crisis

hyperventilation

hypotension

hypovolemia

hypovolemia / shock

hypoxemia

hypoxia

inhalant related disorders

inhalation injury (toxic gas)

intracranial hemorrhage

metabolic shock

neurogenic shock

non-st elevation myocardial infarction (nstemi)

orthostatic hypotension

other cardiovascular disorder

other tracheostomy complication

palpitations

pneumonia

pneumothorax

primary pulmonary hypertension

pulmonary edema, acute

pulmonary embolism

respiratory arrest

respiratory condition due to chemicals, gases, fumes, and vapors

respiratory disorder

respiratory failure

respiratory shock

sepsis/septicemia

septic shock

severe acute respiratory syndrome (sars)

shortness of breath

smoke inhalation

st elevation (stemi) myocardial infarction of anterior wall

st elevation (stemi) myocardial infarction of inferior wall

st elevation (stemi) myocardial infarction of other sites

st elevation myocardial infarction (stemi)

stroke

subarachnoid hemorrhage (non-traumatic)

subdural hemorrhage (non-traumatic)

suffocation or asphyxia

tracheostomy hemorrhage

tracheostomy obstruction

tracheostomy problem

transient cerebral ischemic attack (tia)

**Seizure**

convulsions

febrile seizures

seizure

seizures with status epilepticus

seizures without status epilepticus

**Other**

abuse/neglect, suspected

adrenocortical insufficiency

adult general exam, no finding

allergic reaction

altitude sickness

angioedema

aphagia

aphasia

apparent life threatening event

ascites

bedridden

birth injuries to the newborn

brief resolved unresponsive episode

cancer

child general exam, no finding

congenital deformity

contact with venomous animal

contact with venomous plant

decompression sickness

dehydration

dizziness

dysarthria

dystonic reaction

ear problem

edema

effects of air pressure and water pressure

encounter for aftercare

encounter, feared condition not observed

epistaxis

exam/observation for other suspected conditions ruled out

failure to thrive (adult)

failure to thrive (child)

fatigue

foreign body in ear

general exam

general symptoms

generalized edema

heat exhaustion

hematoma (non-traumatic)

hyperglycemia (not diabetic)

hypertension

hypoglycemia (not diabetic)

hypokalemia

hyponasality

hypothermia

jaundice

liver failure

malaise

meconium aspiration

medical device failure

metabolic disorder

muscle weakness

near syncope

need for continuous medical supervision

neonatal withdrawal symptoms from maternal substance abuse

no complaints or injury/illness noted

obesity

obvious death

patient assist only

pitting edema

post-term newborn

pressure ulcers

psychogenic shock

rash

renal failure

respiratory distress of newborn

sunburn

syncope / fainting

thyroid disorder

**GI/GU**

acute abdomen

constipation

diarrhea

displacement of urinary catheter

displacement of vascular dialysis catheter

dysphagia

end stage renal disease (esrd)

esophagitis

esophageal obstruction

foreign body in anus and rectum

foreign body in genitourinary tract, part unspecified

foreign body in larynx

foreign body in vulva and vagina

foreign body of alimentary tract, part unspecified

foreign body on external eye

gastro-esophageal reflux disease (gerd)

gastrointestinal hemorrhage

gastrostomy hemorrhage

gastrostomy infection

gastrostomy malfunction

hematemesis

intestinal obstruction

leakage of vascular dialysis catheter

melena

nausea

urinary system disorder

urinary tract infection (uti)

vomiting

**CNS/Neuro**

bell's palsy

generalized weakness

hemiparesis

hemiplegia

paralysis

paraplegia

quadriplegia

reduced mobility

visual disturbance

**OB/GYN**

ectopic pregnancy

encounter for full-term uncomplicated delivery

immediate postpartum hemorrhage

labor and delivery complications

labor and delivery, uncomplicated

postpartum hemorrhage

pre-eclampsia

pregnancy complications

pregnancy related conditions

pregnancy with contractions

preterm labor with preterm delivery

preterm labor without delivery

preterm newborn

retained placenta without hemorrhage

spontaneous abortion (miscarriage)

vaginal hemorrhage

vomiting due to pregnancy

**Diabetic/Endocrine**

diabetic hyperglycemia

diabetic hypoglycemia

**Cardiac Arrest**

cardiac arrest

**Infection/Immune**

acute bronchitis

acute epiglottitis

cellulitis

common cold

cough

covid-19 - confirmed by testing

covid-19 - exposure to confirmed patient

covid-19 - suspected - no known exposure

encephalitis/encephalomyelitis

fever

health hazard contact /suspected exposure

infectious disease

influenza

laryngitis/croup

meningitis

monkeypox

respiratory syncytial virus (rsv)

skin infection

tracheostomy infection

**Appendix Table A2. Patient-Level Odds Ratios for Very-High EMS Utilization**

| **Variable** | **Univariable Odds Ratio** | **95% Confidence Intervals** |
| --- | --- | --- |
| **Sex** |  |  |
| Female | Referent | — |
| Male | 0.91 | 0.89-0.94 |
| **Race/Ethnicity** |  |  |
| White, not Hispanic/Latino | Referent | — |
| American Indian/Alaskan Native | 0.74 | 0.59-0.93 |
| Asian | 0.08 | 0.05-0.11 |
| Black or African American | 1.42 | 1.37-1.46 |
| Native Hawaiian or Other Pacific Islander | 0.12 | 0.06-0.26 |
| Hispanic/Latino | 0.38 | 0.35-0.41 |
| Multiracial | 5.52 | 5.29-5.75 |
| **Age Categories** |  |  |
| Adult (18-64) | Referent | — |
| Older Adults (≥65) | 0.87 | 0.85-0.89 |
| Adolescent (13-17) | 0.08 | 0.06-0.09 |
| School age (5-12) | 0.05 | 0.03-0.07 |
| Toddler/Preschool (1-4) | 0.02 | 0.01-0.04 |
| Baby (<1 year) | 0.02 | 0.01-0.03 |
| **Index Visit Clinical Impression** |  |  |
| Cardio/Pulmonary | Referent | — |
| AMS/Behavioral/Psych | 0.86 | 0.82-0.90 |
| CNS/Neuro | 0.93 | 0.88-0.99 |
| Seizure | 0.94 | 0.88-1.01 |
| Diabetic/Endocrine | 1.93 | 1.79-2.09 |
| Injury or Pain | 0.62 | 0.60-0.65 |
| Infection/Immune | 0.93 | 0.85-1.01 |
| GI/GU | 1.12 | 1.05-1.19 |
| OB/GYN | 0.22 | 0.16-0.29 |
| Other | 0.63 | 0.61-0.66 |
| **Index Visit REMS** | 0.97 | 0.97-0.98 |

*Notes: Odds ratios compare patients with very-high utilization (12+ encounters) to patients with a single encounter. Analysis conducted at the patient-level(n=6,604,938). REMS = Rapid Emergency Medicine Score.*

**Appendix Table A3. Incident Characteristics**

| Variable Name  %  (n) | All Encounters 100.00% (9,508,809) | Patients with 1 Encounter 56.41% (5,363,570) | Patients with 2-4 Encounters 27.21% (2,587,480) | Patients with 5-11 Encounters 11.14% (1,059,250) | Patients with 12+ Encounters 5.24% (498,509) | Missing |
| --- | --- | --- | --- | --- | --- | --- |
| CDC SVI |  |  |  |  |  |  |
| Quartile 1  (least vulnerable) | 19.15%  (1,788,291) | 20.76% (1,091,607) | 19.06% (485,036) | 15.26% (159,087) | 10.67% (52,561) | 171,184 |
| Quartile 2 | 24.09%  (2,248,982) | 24.70% (1,298,453) | 24.26% (617,343) | 22.66% (236,317) | 19.66% (96,869) |  |
| Quartile 3 | 27.65%  (2,581,885) | 27.16% (1,427,732) | 28.10% (715,174) | 28.83% (300,652) | 28.07% (138,327) |  |
| Quartile 4  (most vulnerable) | 29.11%  (2,718,467) | 27.38% (1,439,210) | 28.59% (727,521) | 33.25% (346,664) | 41.61% (205,072) |  |
| Community Size | | | |  |  |  |
| Urban | 80.66%  (7,651,010) | 81.79% (4,378,244) | 79.05% (2,039,392) | 77.99% (823,238) | 82.41% (410,136) | 23,123 |
| Rural | 16.75%  (1,589,005) | 15.48%  (828,839) | 18.33% (472,852) | 19.68% (207,697) | 16.00% (79,617) |  |
| Super Rural | 2.59%  (245,671) | 2.72%  (145,627) | 2.62% (67,500) | 2.33% (24,597) | 1.60% (7,947) |  |
| EMS Transport Disposition | | | | |  |  |
| Transported by EMS | 79.10%  (7,521,032) | 75.17% (4,031,979) | 84.45% (2,185,035) | 84.04% (890,154) | 83.02% (413,864) | N/A |
| Non-Transport | 20.40%  (1,940,253) | 24.11% (1,293,073) | 15.25% (394,657) | 15.86% (168,023) | 16.95% (84,500) |  |
| Dead on Scene | 0.50%  (47,524) | 0.72%  (38,518) | 0.30% (7,788) | 0.10% (1,073) | 0.03% (145) |  |

**Appendix Table A4. Payer Status and Hospital Outcomes Among Transported Patients with Linked ED Data**

| **Variable%(n)** | **All Encounters** | **Patients with 1 Encounter** | **Patients with 2-4 Encounters** | **Patients with 5-11 Encounters** | **Patients with 12+ Encounters** |
| --- | --- | --- | --- | --- | --- |
| **Total with Linked Data** | 2,345,496 (100%) | 1,203,221 (51.3%) | 692,717 (29.5%) | 297,996 (12.7%) | 151,562 (6.5%) |
| **ED Disposition*** | | | | | |
| Discharged from ED | 60.27% (1,413,690) | 63.69% (766,290) | 52.93% (366,675) | 56.05% (167,034) | 75.01% (113,691) |
| Admitted | 32.56% (763,782) | 29.72% (357,580) | 38.76% (268,472) | 35.91% (107,001) | 20.27% (30,729) |
| ED Death | 0.56% (13,065) | 0.83% (10,046) | 0.37% (2,573) | 0.13%  (402) | 0.03%  (44) |
| Transferred | 6.37% (149,519) | 5.52% (66,473) | 7.69% (53,290) | 7.68% (22,876) | 4.54% (6,880) |
| Still a Patient | 0.23% (5,440) | 0.24% (2,832) | 0.25% (1,707) | 0.23%  (683) | 0.14%  (218) |
| **Payer Status**** | | | | | |
| Commercial | 25.38% (595,196) | 30.87% (371,469) | 20.31% (140,698) | 17.76% (52,914) | 19.87% (30,115) |
| Medicaid | 13.46% (315,804) | 12.70% (152,813) | 11.93% (82,669) | 14.82% (44,175) | 23.85% (36,147) |
| Medicare | 15.29% (358,576) | 13.67% (164,529) | 18.75% (129,858) | 16.09% (47,938) | 10.72% (16,251) |
| Veterans Affairs | 0.89% (20,941) | 1.01% (12,165) | 0.81% (5,616) | 0.73% (2,183) | 0.64%  (977) |
| Workers Compensation | 0.33% (7,824) | 0.60% (7,198) | 0.08%  (578) | 0.01%  (42) | 0.00%  (6) |
| Self-Pay | 1.36% (31,925) | 1.84% (22,139) | 0.91% (6,293) | 0.71% (2,123) | 0.90% (1,370) |
| Multiple Payers | 37.72% (884,770) | 31.65% (380,838) | 43.38% (300,482) | 47.14% (140,474) | 41.55% (62,976) |
| Other | 0.11% (2,617 ) | 0.11% (1,360) | 0.12%  (856) | 0.11%  (322) | 0.05%  (79) |
| **Hospital Disposition***** | |  |  |  |  |
| Discharged | 62.35% (429,682) | 64.09% (206,852) | 57.81% (139,867) | 63.11% (60,977) | 78.99% (21,986) |
| Died | 3.94% (27,128) | 5.42% (17,487) | 3.31% (8,007) | 1.52% (1,466) | 0.60% (168) |
| Transferred | 33.64% (231,825) | 30.41% (98,166) | 38.81% (93,897) | 35.30% (34,104) | 20.33% (5,658) |
| Still a Patient | 0.08%  (554) | 0.08% (271) | 0.08%  (184) | 0.08%  (78) | 0.08%  (21) |

*ED Dispositions were available for 31% (2,345,496/7,521,032) of Transported Patients

**Payer status was available for 95% (2,217,653/2,345,496)of patients with linked ED Dispositions

***Hospital Dispositions available for 90% (689,189/763,782) of Admitted Patients

**Appendix Table A5. Odds Ratios for Very-High EMS Utilization by Payer Status**

| **Payer Status** | **Univariable Odds Ratio** | **95% Confidence Intervals** |
| --- | --- | --- |
| Commercial | Referent | — |
| Medicaid | 3.11 | 3.07-3.13 |
| Medicare | 1.62 | 1.60-1.64 |
| Veterans Affairs | 1.47 | 1.42-1.52 |
| Workers' Compensation | 0.44 | 0.41-0.48 |
| Self-Pay | 0.74 | 0.73-0.76 |
| Multiple Payers | 1.74 | 1.72-1.76 |
| Other | 1.06 | 0.99-1.14 |

*Notes: Odds ratios compare patients with very-high utilization (12+ encounters) to patients with a single encounter. Analysis restricted to encounters with linked payer data (n=2,217,653). Patients with encounters documented under multiple payer types were classified as "Multiple Payers”*

**Appendix Table A6. Comparison of Encounters with and without Linked ED Data**

| **Characteristic** | **Encounters with Linked ED Data (n=2,345,496)** | **Encounters without Linked ED Data (n=5,175,536)** |
| --- | --- | --- |
| **Community Size** |  |  |
| Urban | 91.6% | 77.1% |
| Rural | 7.4% | 20.2% |
| Super Rural | 1.0% | 2.7% |
| **CDC Social Vulnerability Index** | | |
| Quartile 1  (least vulnerable) | 18.5% | 19.4% |
| Quartile 2 | 23.2% | 24.5% |
| Quartile 3 | 26.7% | 28.0% |
| Quartile 4  (most vulnerable) | 31.6% | 28.3% |
| **EMS Utilization Category** | |  |
| 1 encounter | 51.3% | 59.3% |
| 2-4 encounters | 29.5% | 25.9% |
| 5-11 encounters | 12.7% | 10.3% |
| 12+ encounters | 6.5% | 4.5% |
